# Round the Hospitals

**Published:** 1917-07-28

**Authors:** 


### ROUND THE HOSPITALS.

THE authorities of St. Bartholomew's Hospital have had under their consideration the exercise of economy generally throughout the hospital. A request to the Treasurer and Almoners is contemplated to endeavour to reduce the present consumption of bread by the nursing and domestic staffs, so far as is consistent with health and the Food Controller's suggestions. The appointment of a Food Supervisor is also contemplated, we understand, at a salary of £350 per annum.

THE twenty-seventh annual report of the Council of the Queen Victoria Jubilee Institute for Nurses, and of the Executive Committee of the Queen's Fund, for 1916 has been issued. The work of the Institute has had again to be carried on under extremely difficult conditions, whilst the year 1916 has been remarkable for the great and increasing activity in all branches of health work. District nurses naturally take a large share in such work, and especially in the schemes for maternity and child welfare, owing to the great impetus these have received from the grants promised by the L.G.B. and the Board of Education. In London special courses of lectures on infant-care work have been arranged for Queen's Nurses, to be followed later on by similar lectures in other parts of the country. Five hundred and eighty-nine Queen's Nurses are engaged away from their work in connection with the war. Fifteen Queen's Nurses, whose names have already been published, have been awarded the Royal Red Cross. A silver Medal of Honour for devoted services to the French soldiers has been presented to Miss E. Ubsdell. The French Government has awarded a medal to Miss E. Patrick, and the silver Médaille d'Honneur des Epidémies to Miss Turnell. Four Queen's Nurses —Miss Ada Stanley, Miss Mary Burt, Miss Jessie Paterson, and Miss S. E. Butler—have given their lives in their country's service. The Council properly insist on the fact that the nurses who have stayed at home are all doing in several ways real service to their country.

A MOST serious consequence of the war to the Jubilee Institute's direct work is the almost complete cessation in the supply of hospital-trained nurses entering for district work. 1916, in consequence, has been a very critical year for the training homes and the Institute, which latter relies on the nurses who are trained under agreement to fill most of the vacancies from time to time. The salaries of the Queen's Nurses have been increased to a minimum of £35, rising to £40. Where an inclusive salary is given the commencing rate is £100, with the usual increases. Large allowances to cover the cost of board and laundry, owing to the rise in prices, amounting to 15s. a week, have been granted. The Institute is now giving a war bonus of £2 10s. to English and Welsh candidates on the completion of six months' training.

DURING the year the Queen's and Village Nurses have attended 35,691 cases of labour, either as midwives or as maternity nurses under the doctors, which the statistics prove to have been most satisfactory. The number of Queen's Nurses working at the end of 1916 was 2,104. The total number of nurses in connection with the Institute, including Queen's Nurses, those in training, village nurses, and midwives was 4,106—*i.e.* England, 3,138; Scotland, 467; Ireland, 204; Wales, 297. One hundred and forty-four women were placed on the roll of Queen's Nurses in 1916. Fifty-one former Queen's Nurses have rejoined and 219 have resigned from the Institute. The total receipts amounted to

£10,805, and the total expenditure to £9,483. The general capital, including all funds, approaches £200,000. The list of places where there are nursing associations employing Queen's Nurses within the United Kingdom fills eighteen demy octavo pages. The work of the Queen Victoria Jubilee Institute is wonderfully well done. It has conferred signal benefit on the humbler residents throughout His Majesty's dominions, and has proved a tower of strength and comfort to the poorer residents not only in large cities, but in small rural communities throughout the land.

---

A NEW Maternity Home has been opened by the Countess of Beauchamp in Russell Street, Gloucester. It is a development of the Gloucester District Nursing Society, and will provide accommodation for difficult, complicated confinement cases occurring in the city and county. Admission is obtainable through the recommendation of a practitioner of medicine. It is contemplated to co-operate with the Gloucester Mothers' Club and Babies' Welcome, and to admit expectant mothers who are found to require hospital treatment. The immediate need of such a maternity ward, especially at the present time, has been felt in the city, where strenuous efforts are being made for the preservation of infant life. Mr. W. B. Wood, architect, is responsible for the structural alterations, which cost £500, the cost of furnishing and equipment is £200, and a sum of £350 is still required to free the Home from debt.

---

THE report of the Community of Nursing Sisters of St. John the Divine for 1916 might, in future years, be enlarged with advantage to the Community, and also to its branch at St. John's Hospital, Lewisham, by a recital of the actual work done by the latter, which would add to the interest of the report. Throughout a long life we have had experience of the work of the Nursing Sisters of St. John the Divine, and we can testify out of fulness of knowledge that any sick person who has the good fortune to be entrusted to the care of a Nursing Sister of this Order is entitled to be regarded as a most fortunate member of the human race. The work done by the sisters has always been supremely good, and the spirit which animates them in the discharge of their duties to the sick is an example to be followed by every trained nurse of the highest standards, who recognises the paramount importance to the sick of the missionary spirit in the nurse. Residents in the neighbourhood of Drayton Gardens, South Kensington, are therefore to be congratulated upon the special opportunities afforded to them in the day of sickness to obtain the services of a Nursing Sister. The Superior makes a good point in her report in urging the special claim for funds which the Community of Nursing Sisters of St. John has upon the public, seeing that the high efficiency of the training of the nurses employed has enabled them, in the absence of many members of the medical profession abroad, to provide for the well-being of the people in the very poor neighbourhoods, where the work and the need are greatest.

---

THE report's exiguity is regrettable in the sense that it has been very difficult indeed for a trained and experienced reader of such documents, after some study, to glean even an approximate view of the character of the work and the position of the Community and its hospital. There is really nothing to guide one to grasp the causes which have created a deficiency in the accounts. We are confident that if the Superior and those responsible for the hospital were candidly to set forth the excellence and character of the work done, its amount, the relative number of poor cases nursed in their own homes, in private families, and in the hospital at Lewisham, some telling statements should result. These when published should produce without difficulty or delay the relatively small sum (under £500) which is required to place this excellent Community and its organisation upon a sound financial basis.

---

**The "Choker" Collar in Red Cross Nursing.**

SIR,—As you invite the expression of views on the subject of nursing uniform, I venture to give mine. I trained twenty-five years ago, when we wore a narrow-linen collar, and even that was uncomfortable. Now, after nearly three years of matronship in Red Cross hospitals, I cannot protest too strongly against the "choker" that the modern nurse wears. I find the probationers unfasten theirs when doing any hard or bending work, such as bed-bathing, etc., and the sisters give as their reason for not spending off-duty time in the gardens, that they cannot take off their collars, and that is the first thing they must do on leaving the wards. Can anything be more ridiculous than such an uncomfortable adjunct to a working dress? My V.A.D.M.s now wear a turn-down, unstarched collar—the Ranelagh—and look very smart, but it must be properly worn with the dress collar turned in and the apron-bib coming right up to the neck, so that collar and bib look to be one garment. As long as any uniform is clean and worn smartly, there need be no fear of loss of prestige or authority.—Yours, etc.,

"A RED CROSS MATRON."

[If the "choker" collar is really as bad as it is painted in this letter from a matron of experience and reputation, we hope the Red Cross authorities will end it or mend it without a moment's avoidable delay.—ED. THE HOSPITAL.]

---

*** It is our invariable practice to pay for all items of news which we may publish. The minimum payment is 5s. Paragraphs of about twenty lines in length—that is to say, of 150 words or less—are preferred. In this section during the war we propose to give 10s. to the correspondent who may send us in any week the best incident connected with hospital life and work. In this way we hope to afford to all practical aid and encouragement; while those who reciprocate will be able to render us invaluable service. Contributions should be addressed to The Editor, THE HOSPITAL, 28 and 29 Southampton Street, Strand, London, W.C., and, to be in time for the current week's issue, should reach him by the first post on Mondays.

